# Epididymal adrenal rest in an orchiectomy specimen with seminoma

**DOI:** 10.4322/acr.2023.464

**Published:** 2023-12-15

**Authors:** Luca Ventura, Giulia Del Rosario, Martina Di Franco, Veronica Forte, Massimo Menichini, Guido Ranieri

**Affiliations:** 1 San Salvatore Hospital, Division of Pathology, L’Aquila, Italy; 2 San Salvatore Hospital, Division of Nuclear Medicine, L’Aquila, Italy; 3 San Salvatore Hospital, Division of Urology, L’Aquila, Italy

**Keywords:** Adrenal Cortex, Epididymis, Orchiectomy, Testicular Neoplasms, Testis

Ectopic adrenal tissue is rare but is reported in various locations within the urogenital tract and abdominal structures. The vast majority of adrenal rests represent incidental findings in surgical specimens; thus, their incidence is unknown.^1^ Notwithstanding, the results of reports on their higher frequency in infants than adults and sex distribution are conflicting.^1,2^ In male subjects, the paratesticular and inguinal regions represent common sites of ectopic adrenal tissue, given the intimate embryologic relationship between the gonad and the adrenal cortex.^3^ In testis and paratestis, they are also known as Marchand rest and are most commonly found in the spermatic cord,^3^ followed by testis^4^ and epididymis.^5,6^ In these anatomic locations, ectopic adrenals may be associated with undescended testis, inguinal hernia, epididymal abnormality, and spermatic cord torsion, but none represent a predisposing factor. Also, the association with malignant testicular neoplasms merely represents a matter of chance. As a rule, adrenal rests do not show significant clinical implications. However, they may undergo hyperplasia when the function of the main adrenals is deficient or in congenital adrenal hyperplasia (CAH), an autosomal recessive disease with increased ACTH levels.^5,6^ Also, adrenal rests may be accidentally removed during surgery, leading to adrenal insufficiency. Finally, ectopic adrenal may harbor benign or malignant tumors resulting in clinically evident dysfunctions.^3^ The adrenal rests comprise nodules ranging between 1 mm and 1 cm, appearing as encapsulated or well-circumscribed round yellowish masses that may be multiple or bilateral. Microscopic appearance reminds normal adrenal cortex, often arranged in different zones, without medullary tissue.

**Figure 1:**
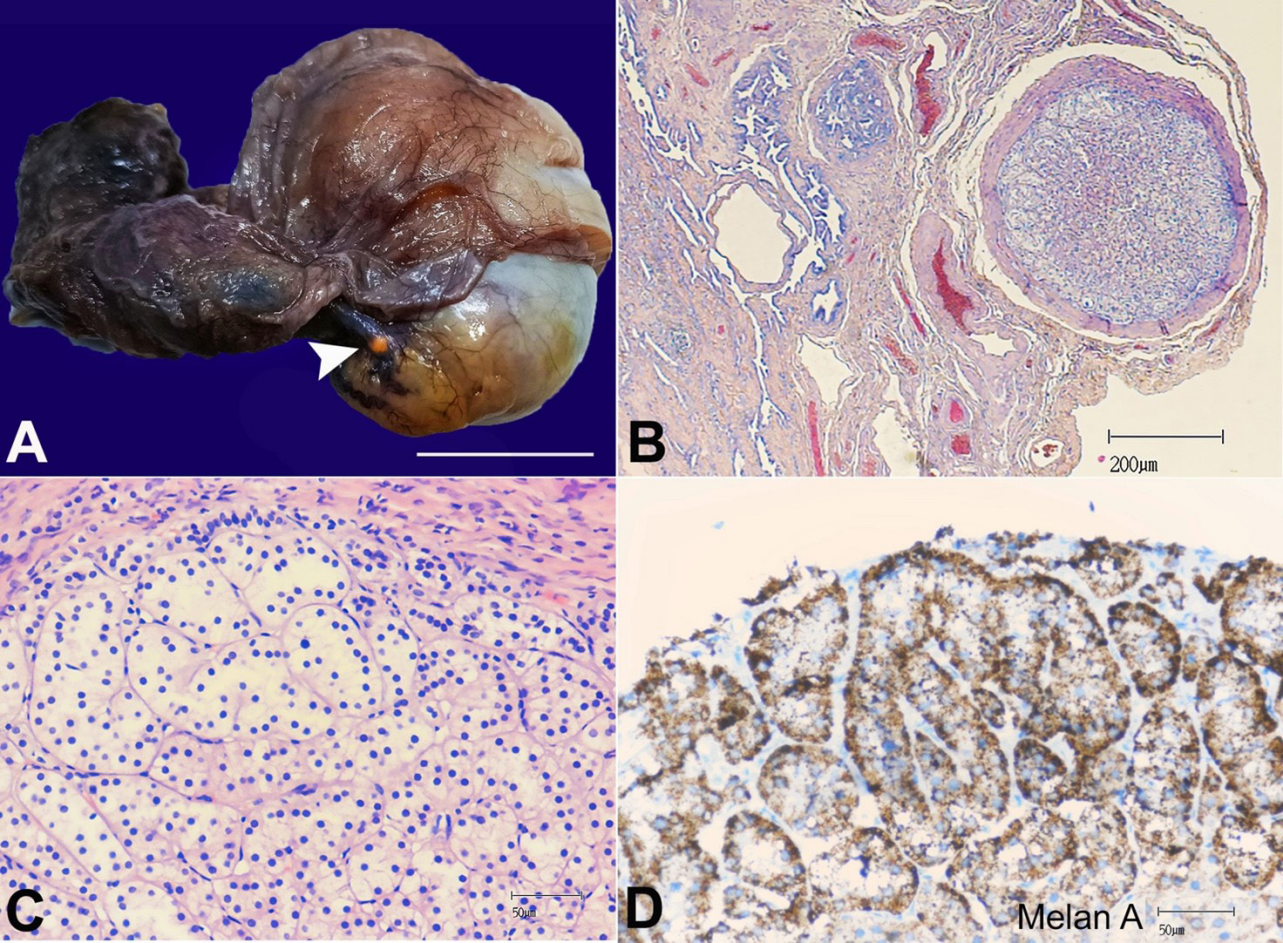
**A -** Gross orchiectomy specimen displaying a tiny nodule yellowish-brown in color (arrowhead) below the visceral layer of the tunica vaginalis and close to the head of the epididymis (scale bar= 3 cm); **B -** Encapsulated adrenal rest located between epididymis and rete testis; **C -** Encapsulated adrenal cortical tissue; **D -** Immunohistochemical positivity for Melan-A.

Figure 1 refers to a case of a tiny adrenal rest nodule incidentally observed in an orchiectomy specimen obtained from a 40-year-old man affected by a suspect germ cell tumor of the right testis. The surgical specimen´s gross examination depicted a yellowish-brown nodule measuring 2 mm in its longest axis, located under the visceral mesothelium of the tunica vaginalis near the head of the epididymis (Figure 1A).

Microscopical examination diagnosed a pure testicular seminoma infiltrating the albuginea and the visceral part of the tunica vaginalis (pT2). Histology showed a well-encapsulated nodule between the epididymis head and the rete testis (Figure 1B). The nodule was composed of epithelial cells arranged into an organoid pattern consistent with the adrenal cortex (Figure 1C). No adrenal medullary tissue was present. The cells were immunohistochemically positive for Melan-A monoclonal antibody (clone A103) (Figure 1D). No immunostaining was observed for inhibin α (clone R1), calretinin (clone DAK-Calret 1), and BCL2 oncoprotein (clone 124).
